# Hydrophobization of Cold Plasma Activated Glass Surfaces by Hexamethyldisilazane Treatment

**DOI:** 10.3390/molecules29112645

**Published:** 2024-06-04

**Authors:** Konrad Terpiłowski, Michał Chodkowski, Evgeniy Pakhlov, Sylwia Pasieczna-Patkowska, Marcin Kuśmierz, Seitkhan Azat, Salvador Pérez-Huertas

**Affiliations:** 1 Department of Interfacial Phenomena, Institute of Chemical Sciences, Faculty of Chemistry, Maria Curie Skłodowska University, Maria Curie Skłodowska Sq. 2, 20-031 Lublin, Poland; m.chodkowski@pollub.pl (M.C.); pexim@ukr.net (E.P.); 2 Department of Technology and Polymer Processing, Faculty of Mechanical Engineering, Lublin University of Technology, ul. Nadbystrzycka 36, 20-618 Lublin, Poland; 3 Chuiko Institute of Surface Chemistry, National Academy of Sciences of Ukraine, 03164 Kyiv, Ukraine; 4 Department of Chemical Technology, Institute of Chemical Sciences, Faculty of Chemistry, Maria Curie Skłodowska University, Maria Curie Skłodowska Sq. 2, 20-031 Lublin, Poland; sylwia.pasieczna-patkowska@mail.umcs.pl; 5 Analytical Laboratory, Institute of Chemical Sciences, Faculty of Chemistry, Maria Curie Skłodowska University, Maria Curie Skłodowska Sq. 2, 20-031 Lublin, Poland; marcin.kusmierz@mail.umcs.pl; 6 Satbayev University, 22a Satbayev Str., Almaty 050013, Kazakhstan; seytkhan.azat@gmail.com; 7 Department of Chemical Engineering, University of Granada, Av. de Fuente Nueva s/n, 18071 Granada, Spain

**Keywords:** hexamethyldisilazane, hydrophobization, plasma, glass, wettability, surface properties

## Abstract

The objective of this study was to investigate the modification of glass surfaces by the synergistic combination of cold plasma and chemical surface modification techniques. Glass surface hydrophobicity was obtained as a result of various plasma and deposition operational conditions. The mechanisms governing the hydrophobization process were also studied. Glass plates were activated with plasma using different gases (oxygen and argon) at different treatment times, ranging from 30 to 1800 s. Then, the plasma-treated surfaces were exposed to hexamethyldisilazane vapors at different temperatures, i.e., 25, 60, and 100 °C. Complete characterization, including contact angle measurements, surface free energy calculations, 3D profilometry, X-ray photoelectron spectroscopy, Fourier-transform infrared spectroscopy, and scanning electron microscopy, was accomplished. It was found that the extent of the hydrophobicity effect depends on both the plasma pre-treatment and the specific conditions of the hexamethyldisilazane deposition process. Plasma activation led to the formation of active sites on the glass surface, which promoted the adsorption and reaction of hexamethyldisilazane species, thereby inducing surface chemical modification. Longer plasma pre-treatment resulted in stronger modification on the glass surface, resulting in changes in the surface roughness. The largest water contact angle of ≈100° was obtained for the surface activated by argon plasma for 1800 s and exposed to hexamethyldisilazane vapors at 25 °C. The changes in the surface properties were caused by the introduction of the hydrophobic trimethylsilyl groups onto the glass surface as well as roughness development.

## 1. Introduction

Surface hydrophobization is a fundamental process that involves the modification of surface properties of a material, resulting in a reduction in its wetting by water. The precise control of this process is crucial for a wide range of industrial applications in such fields as aerospace [1], textile [2], construction [3], biomedical devices [4] etc. The surface wettability is influenced by both its morphology and surface free energy (SFE). Several authors have experimentally demonstrated that surface hydrophobicity is significantly affected by both vertical-perspective roughness factors (denoted as f in the Cassie-Baxter model [5], and r in the Wenzel model [6]) as well as horizontal-perspective roughness factors, such as the spacing between surface irregularities and the shape of those irregularities [5,6,7]. Thus, two main strategies can be applied to induce hydrophobicity in a given material, i.e., reducing its surface free energy and tailoring the surface roughness. One of the most commonly used methods for reducing SFE is surface chemical modification. This process involves the use of chemicals with low SFE, which undergo a surface reaction, leading to the introduction of hydrophobic groups onto the treated surface. For instance, Chen et al. [8] hydrophobized capacitive humidity sensors based on silicon nanowires using hexamethyldisilazane (HMDS). The nanowires were exposed to HMDS vapors at 120 °C for 20 min, which resulted in a chemical reaction where hydroxyl surface groups were substituted by trimethylsilyl (TMS) ones [9]. The HMDS treatment modified the surface nature of the sensor from hydrophilic to hydrophobic, reducing the water vapor adsorption and thus improving the sensor performance. Furthermore, the incorporation of the hydrophobic group can be enhanced by tailoring the surface topography of the material.

Cold plasma is a state of matter composed of partially ionized gas at low temperatures. It is characterized by the presence of ions, electrons, neutral particles, and reactive species [10]. Using low SFE agents to generate plasma, cold plasma treatment can introduce hydrophobic groups onto the treated surface, inducing a strong hydrophobic character in the material [11,12]. Another noteworthy capability of cold plasma treatment is its ability to tailor the surface roughness of materials [13]. By adjusting the parameters of the plasma treatment, e.g., gas composition, power, or treatment time, it is possible to control the morphology of the treated surface and induce roughness at the micro- and nanoscale levels [14]. Additionally, this kind of treatment can generate active sites on the substrate surface, which facilitates subsequent adsorption and reaction or deposition of hydrophobic chemicals [15]. Consequently, the use of cold plasma prior to the chemical modification techniques will enable better control of the hydrophobization process. Saito et al. [16] deposited organosilicon compounds (hexamethyldisilazane and hexamethyldisiloxane) on glass, copper, and nickel plates using an atmospheric pressure thermal microplasma jet. The organosilicon compounds were vaporized in argon and the plasma jet was irradiated onto the substrates. It was found that the organosilicon species were successfully deposited on the treated substrates, forming various hydrophobic structures, i.e., cotton-like, irregular, string-like, and flat thin layers. According to the authors, obtaining a specific structure depended on the concentration of organosilanes in the plasma gas and the distance from the nozzle to the substrate surface. Yamamoto et al. [17] used atmospheric pressure nonthermal plasma to activate glass surfaces before their modification with trialkoxysilanes and found a water contact angle (WCA) of 108°. The authors proved that the plasma activation caused changes in the surface chemistry of the treated glass, promoting the formation of hydrophobic Si-O-R structures. Ting et al. [18] produced a composite coating with anti-corrosion and anti-osteoconduction properties combining plasma polymerized HMDS with calcium phosphate. Deposition of the organosilanes layer on the glass surface increased the WCA from 40 to 100° for the untreated and HMDS-treated surfaces, respectively. The author stated that the plasma activation increased the roughness of the treated surface and promoted the adhesion between both layers. In previous studies, Terpiłowski et al. [19] modified fumed silica with HMDS and monitored the degree of hydrophobization by adjusting the silazane concentration. Hydrophobic silica was mixed with a polystyrene solution in chloroform and the resulting dispersion was applied onto the plasma-activated glass plates via spin coating. It was found that the higher the concentration of HMDS during the silica modification, the larger the WCA, reaching up to 150°.

In this paper, the hydrophobicity of glass surfaces was tailored by combining cold plasma pre-treatment and surface chemical functionalization techniques. The impact of activation and deposition conditions, e.g., gas used to generate the plasma, treatment time, and HMDS vapors deposition temperature, on the glass surface properties was investigated. Glass surfaces were characterized by means of contact angle measurements, SFE calculations, 3D profilometry, X-ray photoelectron spectroscopy, Fourier-transform infrared spectroscopy as well as scanning electron microscopy. It should be noted that the experimental design was made considering the future technological and economic aspects of the proposed hydrophobization process. Developing strategies for the precise control of hydrophobicity degree is crucial in numerous applications, including self-cleaning, anti-corrosion, anti-icing, medical devices, water/oil separation, packaging, etc.

## 2. Results and Discussion

### 2.1. Wetting Behaviour

**Figure 1:**
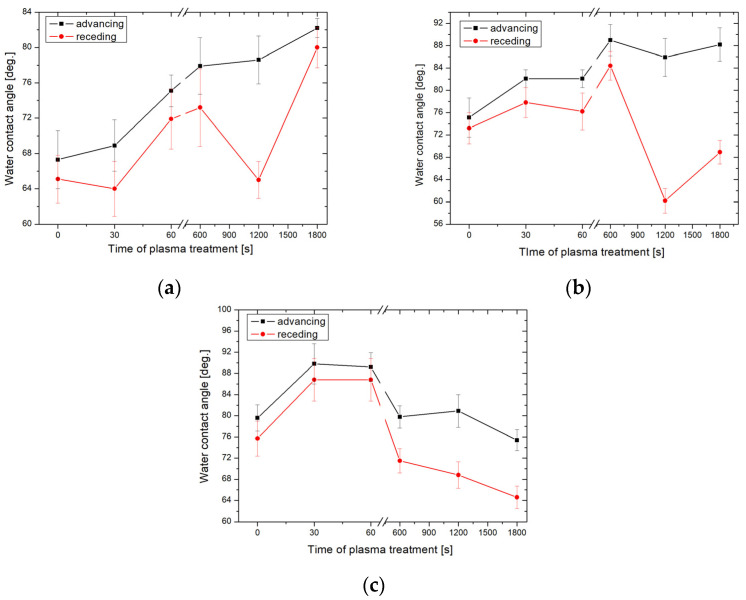
Advancing and receding WCAs obtained on the oxygen plasma-activated glass plates with HMDS vapors deposited at 25 °C (**a**), 60 °C (**b**), and 100 °C (**c**).

Figure 1 shows the advancing WCAs of the glass surface after the oxygen plasma treatment and subsequent exposure to HMDS vapors at varying temperatures.

**Figure 2:**
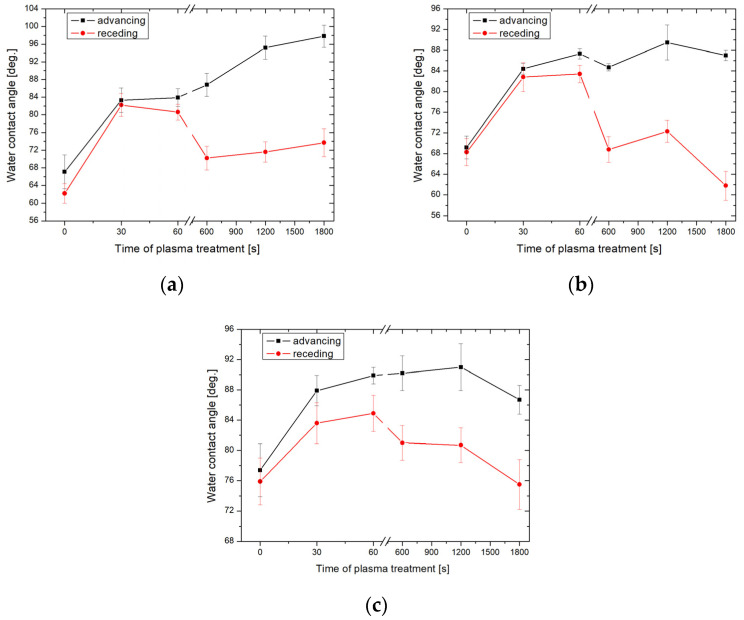
Advancing and receding WCAs obtained on the argon plasma-activated glass plates with HMDS deposited at 25 °C (**a**), 60 °C (**b**), and 100 °C (**c**).

Figure 1 shows that the proposed hydrophobization strategy can tailor effectively the glass surface wettability from hydrophilic (WCA of nonmodified glass is ≈ 30° [20]) to hydrophobic properties. The impact of plasma pre-treatment duration on the sample wettability is evident, especially for the samples exposed to HMDS vapors at 25 and 60 °C. For instance, for the samples exposed to HMDS vapors at 25 °C, the advancing CA increased from 67.3 ± 3.3° to 82.5 ± 2.6° for the non-treated and for the 1800 s plasma pre-treated samples, respectively. Furthermore, this increment shows a nearly linear dependence between the activation time and CA. A similar trend was found for the samples exposed to HMDS vapors at 60 °C (Figure 1b). Even a 30 s oxygen plasma pre-treatment resulted in an increase in advancing CA from 79 ± 2.3° to 90 ± 2.9° (Figure 1c). However, in this case, the CA decreased to its initial values with the increasing pre-treatment time. It should be highlighted that increasing the chemical vapor deposition temperature resulted in an increase in the values of CA. For instance, the advancing CAs of the non-activated plasma samples exposed to HMDS vapors at 25, 60, and 100 °C were 67.3 ± 3.3°, 75.1 ± 3.5°, and 79.6 ± 2.5°, respectively. The largest CA of 90.1 ± 1.8° was obtained for the sample activated for 600 s and exposed to HMDS at 60 °C. Similar findings were reported by Ting et al. [21] who investigated the wettability of silicon treated with plasma polymerized HMDS and observed an increase in the WCA from 54.3° to 95.0°. In another study, Fiorillo et al. [22] obtained thin film transistors by depositing a fullerene-derived semiconductor on the SiO_2_ substrate with the self-assembled HMDS monolayer. Deposition of the functional layers was made at 7, 25, and 60 °C. The largest CA of 108° was obtained when the HMDS deposition was performed at 25 °C. In the cited studies, no significant effect of temperature on the wettability of the obtained coatings was observed. Figure 2 shows the advancing WCA of the glass surface treated by argon plasma and HMDS.

**Figure 3:**
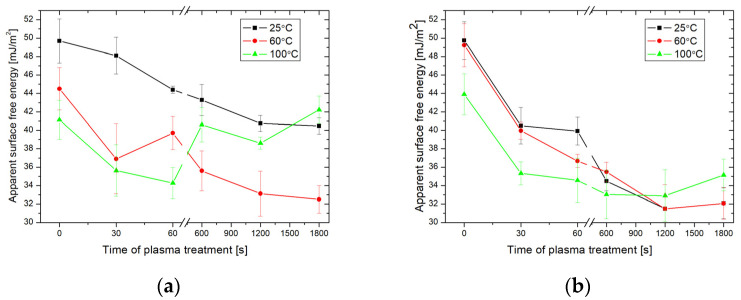
Apparent SFE values based on the CAH approach for the glass surfaces activated by oxygen (**a**) and argon (**b**) plasma as well as modified with HMDS vapors.

Generally, the samples activated with argon plasma showed larger values of CA than those activated by oxygen plasma. Similarly to the previous case, the CA of the argon plasma pre-treated samples increased as a function of the activation time and the temperature during the HMDS exposure. The largest value of the advancing CA of 98.7 ± 2.5° was obtained for the sample activated with argon plasma for 1800 s and subsequently exposed to HMDS vapors at 25 °C. (Figure 2a). Furthermore, a larger CA hysteresis is observed in the case of the samples activated longer than 600 s. The glass surface properties were also determined from the calculations of the apparent SFE. The CAH approach was chosen because it took into account the influence of CA hysteresis on the SFE of the material [23]. Figure 3 shows the apparent SFE calculated based on the CAH hysteresis approach using the measured advancing and receding WCAs.

In general, the SFE decreased with the increasing plasma activation time for most temperatures of the HMDS vapors deposition process. The largest change in the SFE resulting from the plasma activation of the glass surface was obtained with argon plasma. The SFE decreased from 49.3 ± 2.5 to 32.1 ± 1.4 mJ/m^2^ for the samples pre-treated with argon plasma and exposed to HMDS vapors at 25 and 60 °C. The interpretation of the effects of plasma activation on the wettability of the treated glass surface must take into account the amount of the increase in SFE after activation and the nature of the molecules interacting with the surface during the process. It is predicted that plasma pre-treatment (especially oxygen plasma) generated active sites on the glass surface which subsequently interacted with the HMDS vapors and resulted in the formation of new hydrophobic functional groups on the treated surface. If so, XPS and FT-IR spectroscopy should confirm this phenomenon. Therefore, the samples characterized by the greatest values of CA, i.e., the oxygen plasma-activated sample modified with HMDS at 25 °C and the argon plasma-activated sample modified with HMDS at 60 °C, were selected for further analysis.

### 2.2. Surface Morphology

Table 1 shows the surface roughness parameters, R_a_ and R_q_, of non-activated and oxygen or argon plasma pre-treated samples for 1800 s before surface modification with HMDS vapors.

The roughness parameters show that plasma activation of glass plates resulted in an increase in surface roughness, regardless of the type of gas used for plasma generation. The average roughness, R_a_, increased from 3.3 ± 0.7 nm (non-treated glass) to 4.4 ± 0.7 nm and 5.7 ± 0.7 nm for the samples activated by oxygen and argon plasma, respectively. Consequently, the argon plasma treatment produced a rougher surface than the oxygen plasma activation. It is well known that the roughness of a surface influences its wetting behavior. Furthermore, a surface with a greater roughness is likely to enhance the HMDS vapor deposition process. Thus, these results can account for the larger CA and lower SFE observed in the argon-plasma-treated samples. Similar findings can be obtained from the analysis of the other roughness parameters (Table 1). Table 2 and Table 3 show the surface roughness parameters of the samples activated by argon plasma and deposited HMDS vapors at 25 and 60 °C.

**Figure 4:**
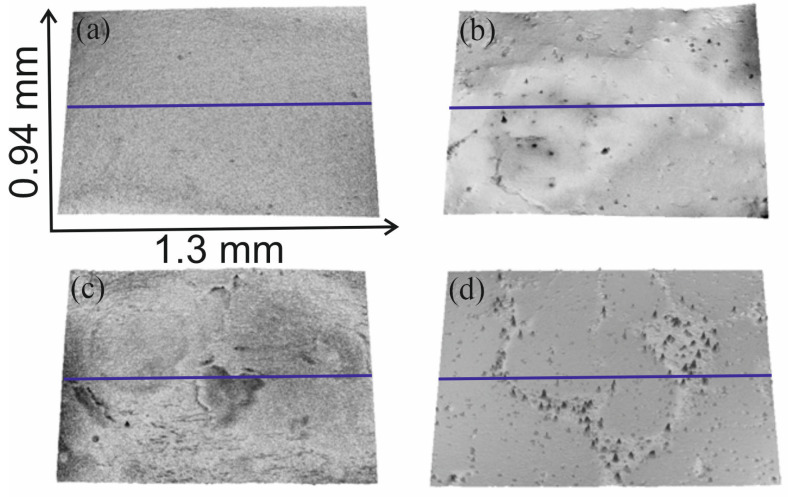
3-D surface height maps of the samples: (**a**) 30 s oxygen plasma pre-treatment and HMDS deposition at 60 °C; (**b**) 1800 s oxygen plasma and HMDS deposition at 60 °C; (**c**) 30 s argon plasma pre-treatment and HMDS deposition at 25 °C; (**d**) 1800 s argon plasma pre-treatment and HMDS deposition at 25 °C (roughness parameters were counted along the blue line visible in the images).

Exposure of plasma pre-treated surfaces to HMDS vapors also induced changes in the surface roughness of the samples. As follows from the comparison of Table 1 with Table 2 and Table 3, R_a_ decreased for all surfaces modified with HMDS, indicating a smoothing effect on the surface after exposure to HMDS vapors. Therefore, the modification resulted in a decrease in surface roughness. This can be explained by the introduction of the hydrophobic groups onto the treated surfaces. Moreover, the deposition temperature also affected the samples’ roughness. The roughness parameters indicate that the surface became rougher for the samples exposed to HMDS at 60 °C and activated for up to 30 s. However, for the samples subjected to longer activations, the surface roughness decreased when exposed to HMDS vapors at 25 °C (Table 2 and Table 3). Additionally, Table 2 and Table 3 show that the longer the plasma activation, the rougher the resulting surface. These changes are also visible in Figure 4, which shows the 3-D surface height maps obtained using the optical profilometer. As can be seen, longer plasma activations resulted in more significant structural changes in the treated surfaces (Figure 4a vs. Figure 4b,c vs. Figure 4d). The samples pre-treated for 30 s (Figure 4a,c) displayed a more uniform surface compared to those pre-treated for 1800 s (Figure 4b,d). Furthermore, the images show that the surfaces treated with argon plasma were rougher, with randomly appearing peaks (Figure 4d). These surface irregularities affected the surface roughness which, in turn, impacted surface wettability.

**Figure 5:**
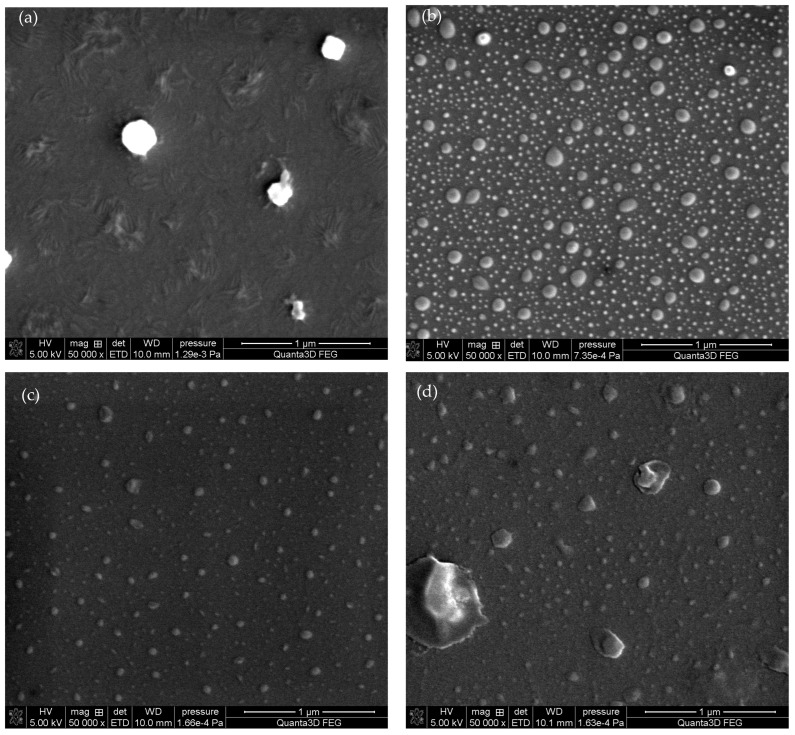
SEM micrographs of the samples pre-treated with 1800 s oxygen plasma and HMDS deposited at 25 °C (**a**) or at 60 °C (**b**), and 1800 s argon plasma and HMDS deposited at 25 °C; (**c**) or at 100 °C (**d**).

In order to investigate the effects of deposition temperature on the glass surface structure, Figure 5 depicts the SEM micrographs of the samples obtained under identical plasma pre-treatment conditions but at different temperatures during the HMDS vapors deposition process.

Figure 5 shows that the temperature affected significantly the surface structure during the HMDS deposition process. In the case of oxygen-treated samples, the deposition of HMDS vapors at 25 °C resulted in the formation of irregular structures (Figure 5a). Moreover, increasing the deposition temperature to 60 °C resulted in the formation of droplet-like structures, with a size smaller than one micrometer (Figure 5b). Similar structures can be observed in the case of argon plasma-treated samples. At a lower temperature, small structures were formed on the glass surface (Figure 5c), whereas at 100 °C, larger clusters were obtained (Figure 5d). The surface morphology reproduced in the SEM micrographs can contribute to the surface roughness presented in Table 2 and Table 3. For instance, Rosales et al. [24] synthesized SiO_2_-TiO_2_ composites by the sol-gel method with the addition of PDMS (polydimethylsiloxane) and HMDS and obtained similar surface structure sizes in the micrometric scale. This allowed us to obtain the superhydrophobic effect consistent with the Cassie-Baxter wettability theory [5].

### 2.3. FT-IR/ATR Spectra

**Figure 6:**
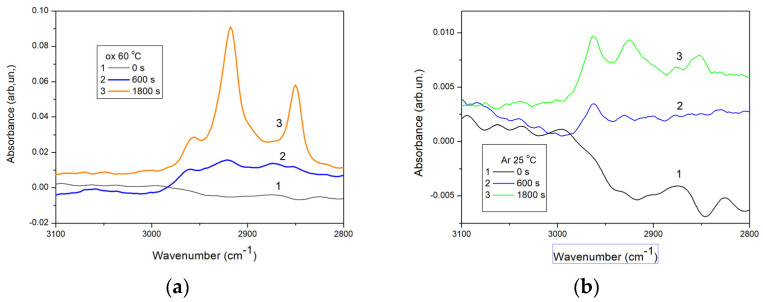
Spectra of glass plates activated with oxygen plasma and HMDS deposited at 60 °C (**a**); and with argon plasma and HMDS deposited at 25 °C (**b**).

To determine the changes in the chemical structure of the modified samples, Figure 6 presents the spectra of the samples treated with oxygen plasma and then exposed to HMDS vapors at 60 °C and those treated with argon plasma and then exposed to HMDS vapors at 25 °C.

As shown in Figure 6, noticeable alterations in the intensity of the bands at the wavenumber of 2853, 2960, and 2960 cm^−1^ are observed for the samples modified with HMDS, corresponding to the stretching vibrations of methyl groups (Figure 6, plots 2 and 3) included in the surface trimethylsilyl groups. Furthermore, the changes in the intensity of these characteristic peaks are more significant for the samples obtained with longer plasma activations (Figure 6, plot 3). The greatest change in the intensity can be observed in the case of the long oxygen plasma pre-treatment. It can be concluded that oxygen plasma modification forms (among others) hydroxyl groups onto the surface [25,26], which subsequently react with HMDS vapors during the hydrophobization process. Kodaira et al. [27] deposited HMDS films onto the glass plates using an atmospheric plasma torch from a mixture of HMDS, argon, and air. The deposition was performed at various times, ranging from 30 s to 2 min. Extending the plasma treatment increased the thickness of the obtained layer, although it did not exceed 2 μm. The largest CA of 80° was obtained. Moreover, the films were deposited on the polyethylene plates for the infrared spectroscopy analysis. The authors identified characteristic peaks, such as the trimethylsilyl group vibrations at 800 and 1260 cm^−1^ as well as the band at 2900 cm^−1^ corresponding to the stretching vibrations of methyl groups. These vibrations were directly associated with the hydrophobic properties of the surface. Moreover, Yang et al. [28] reported similar findings when studying the plasma deposition of HMDS coatings on the TiNi surface to prevent material corrosion. The IR spectra revealed the C-H stretching vibrations in the TMS groups around 2960, 2923, and 2853 cm^−1^ originating from the surface modification with HMDS. The WCA increased from 59° to 90° for the untreated and HMDS modified samples, respectively. In these studies, the increase in the WCA was attributed to the methyl groups included in the TMS moieties incorporated on the surface due to the HMDS treatment. The results reported in the previous studies are consistent with those obtained in this study.

### 2.4. X-ray Photoelectron Spectroscopy

The samples analyzed by FT-IR/ATR were also subjected to X-ray photoelectron spectroscopy (XPS). Table 4 and Supplementary Materials present the results of the XPS analysis performed using the selected samples.

**Figure 7:**
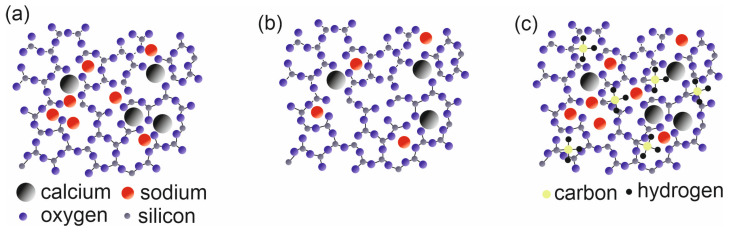
Scheme of the examined materials (**a**) bare glass, (**b**) plasma-activated glass, (**c**) plasma-activated glass with deposited HMDS.

The data presented in Table 4 indicate that the glass used as a sample substrate was a low-sodium one. Regardless of sodium concentration plasma treated glass the surface is chemically active, and can adsorb various chemicals from the environment readily, thus the results obtained using various methods can be inaccurate. Figure 5 shows the structure of embedded HMDS layers. It can be readily visible that this structure is not homogenous. In the other studies [29] the amount of sodium was larger and ranged beginning with 12.1%. It can be seen that after the activation of the plasma on the surface, there is a seemingly active site in which adsorption is promoted. Due to this the glass structure is a supercooled liquid [30] (Figure 7a) which indicates that it is not chemically homogeneous on the surface. The spot, as already mentioned above, has a size of several square millimeters. Nevertheless, conclusions can be drawn from the atomic contribution of individual elements on the surface of the tested samples.

The analysis of XPS spectra is mainly based on the changing proportion of silicon on the surface. It should be remembered that various forms of carbon can adsorb on the surface of the air, especially on the plasma-activated surfaces. On the other hand, silicon is a better choice for the interpretation of the presented results because it could originate from the surface modification with HMDS. As shown in Table 4 and Figure 7b after activating the glass surface with plasma, the amount of sodium and silicon decreases on the surface. In our previous study [20] which used a 1 min activation and a plasma of 160 W, it was not possible to influence the amount of silicon on the surface of the tested material in this way. As follows from the research, increasing the power and extending the time of plasma operation allow to affect the amount of silicon atoms on the surface. After activation, the surfaces become so strongly hydrophilic [20] that it is not possible to measure the contact angles, the only thing that can be measured is the kinetics of wettability which depends on the type of plasma. Data presented in Table 4 indicate that deposition of HMDS contributes to an increase in the surface silicon concentration of all samples. Silicon appears on the surface in the form of -SiCH_3_ groups (Figure 5 and Figure 7c). In the XPS spectrum this corresponds to the peak at 284.2 eV [31]. The spectra of C1s are presented in the Supplementary Materials, (Table S1) as a result of modification with HMDS, the proportion of carbon in the form of -CH_3_ groups increased. Unfortunately, the amounts of introduced HMDS are small, thus peaks corresponding to -SiCH_3_ groups are not visible. As a result of the activation of the plasma substrate surface, some silicon atoms are removed and replaced by the -SiCH_3_ groups (Figure 7c). The evidence of this phenomenon is also the SEM mappings (Figure 7) showing the heterogeneous structure of HMDS layers deposited on the glass surface. Due to the structure of the HMDS layers (Figure 5), the XPS method is a certain approximation and should be treated as a semi-quantitative one.

## 3. Materials and Methods

### 3.1. Plasma Pre-Treatment

Microscopic slides (ChemLand, Stargard, Poland) were placed in the Pico Low-Pressure Plasma System (Diener Electronic, Ebhausen, Germany) for plasma treatment. The chamber was pressurized to 0.2 mbar. The gas flow was controlled by the chamber pressure and vacuum pump and was set at 20 sccm (standard cubic centimeters per minute). The activations were made using plasma generated from both oxygen and argon. The surface was treated for 30, 60, 600, 1200, and 1800 s, with the maximum power of the generator being 1000 W.

### 3.2. Surface Modification

A total of 10 mL of 1,1,1-trimethyl-N-(trimethylsilyl)silanamine (hexamethyldisilazane, HMDS) (99%, Sigma-Aldrich, St. Louis, MO, USA) was placed in the desiccator with a removed bed, and the non-activated and plasma-activated glass plates were put inside. The chemical modification process was conducted at different temperatures, i.e., 25, 60, and 100 °C for 24 h. These conditions were applied based on the boiling point of HMDS (125 °C). Finally, the glass plates were heated at 100 °C for 1 h to remove unbound HMDS residues as well as a by-product.

### 3.3. Contact Angle Measurements

The contact angles (CA) were measured using a GBX Digidrop Contact Angle Meter (GBX, Dublin, Ireland). The measurements were made in a chamber under the controlled conditions of 20 °C and 50% relative humidity. In this case, water purified by the Milli-Q system (Merck Millipore, Burlington, MA, USA) was also used. The droplets were of 6 μL initial volume. After placing a droplet in front of the lens, the advancing CA of both sides was measured using the Digidrop software (version 15.09.07 01 GB, Lyon, France ) with polynomial fitting. In the next stage, the volume of the droplet was reduced by 2 μL, and the receding CA was measured using the same procedure. All results were obtained as the average from 10 measurements.

### 3.4. Surface Free Energy Determination

The apparent surface free energy was calculated using the theory based on the contact angle hysteresis (CAH) proposed by Chibowski [32]:

(1) $$\gamma_{s}^{tot}=\frac{\gamma_{l}{\left(1+\cos\theta_{a}\right)}^{2}}{\left(2+\cos\theta_{r}+\cos\theta_{a}\right)}$$

where: $\gamma_{s}^{tot}$—the apparent SFE, θ_a_—the advancing CA, θ_r_—the receding CA, $\gamma_{l}$—the probe liquid (water) surface tension. The apparent SFE was calculated from at least 10 pairs of advancing and receding contact angles.

### 3.5. Optical Profilometry

The topography of the samples was investigated using an optical profilometer Contour GT 3D Optical Profiler (Bruker Nano Surface Division, Tuscon, AZ, USA). Moreover, the Vision64^®^ software was used for the determination of the roughness parameters: arithmetic mean roughness (R_a_) and root mean square roughness (R_q_). The sampling area was 0.9 × 1.3 mm, approximately equivalent to the surface area occupied by a 6 µL droplet used for the CA measurements.

### 3.6. Scanning Electron Microscopy (SEM)

The morphology of the samples was studied using the SEM technique. The micrographs were taken by means of Quanta 3D FEG (FEI, Eindhoven, The Netherlands) apparatus equipped with the Everhart–Thornley detector (ETD). The beam operated at 5 kV and the other specific parameters are presented in the SEM micrographs.

### 3.7. Fourier-Transform Infrared Spectroscopy

The attenuated total reflectance Fourier-transform infrared (FT-IR/ATR) spectra were recorded in the 4000–400 cm^−1^ range with a resolution of 4 cm^−1^ at room temperature using the Meridian Diamond ATR accessory (Harrick) and Nicolet 6700 spectrometer (Thermo Scientific, Waltham, MA, USA). The samples were applied directly onto the diamond crystal, and close contact with the surface was obtained by the pressure tower. The interferograms of 512 scans were averaged to generate each spectrum. All spectra were corrected for water vapor and carbon dioxide presence, and no smoothing functions were used.

### 3.8. X-ray Photoelectron Spectroscopy (XPS)

The Ultra-High Vacuum analytical system (Prevac, Rogów, Poland), equipped with the Scienta MX 650 X-ray source and the R4000 high-resolution electron energy analyzer (spot 2 × 8 mm) was used to record the XPS spectra in order to study changes in the surface chemical composition of the samples.

## 4. Conclusions

Glass surface hydrophobicity was finely tuned by tailoring the plasma pre-treatment and the HMDS vapors deposition operational conditions. Plasma activation of the glass surface improved significantly the hydrophobization efficiency induced by the chemical modification with HMDS vapors. In most cases, extending the plasma treatment time to more than 30 s, resulted in an increase in the value of the advancing WCA (75–100°) and a decrease in the SFE (40–30 mJ/m^2^). The temperature of the chemical modification process with HMDS vapors also affected the wettability of the samples. However, this effect was less prominent in the case of the samples with long plasma pre-treatment time (>60 s). Furthermore, the SFE of the hydrophobized samples decreased with the increasing plasma activation time. The highest value of contact angle of 98.7° was obtained for the samples activated by argon plasma for 1800 s and exposed to HMDS vapors at 25 °C. The plasma pre-treatment resulted in chemical and physical changes in the surface structure. The change in the surface properties was due to the introduction of the hydrophobic trimethylsilyl groups on the glass surface as well as the formation of a rough structure.

## Figures and Tables

**Table 1:** The roughness parameters of glass surfaces before HMDS vapors deposition.

Sample	Non-Activated	1800 s O_2_ Plasma Treatment	1800 s Ar Plasma Treatment
R_a_ [nm]	3.3 ± 0.7	4.4 ± 0.7	5.7 ± 0.9
R_q_ [nm]	4.3 ± 0.8	5.5 ± 0.8	7.0 ± 1.2

**Table 2:** The roughness parameters of glass plates activated by argon plasma and HMDS vapors deposited at 25 °C.

Pre-Treatment Time [s]	0	30	60	600	1200	1800
R_a_ [nm]	2.0 ± 1.0	2.4 ± 1.0	3.2 ± 0.3	4.1 ± 0.7	5.5 ± 0.5	4.0 ± 0.9
R_q_ [nm]	2.5 ± 1.2	3.0 ± 1.3	4.3 ± 0.5	5.1 ± 0.8	6.9 ± 0.7	7.1 ± 3.7

**Table 3:** The roughness parameters of glass plates activated by argon plasma and HMDS vapors deposited at 60 °C.

Pre-Treatment Time [s]	0	30	60	600	1200	1800
R_a_ [nm]	2.7 ± 0.7	2.5 ± 0.8	2.6 ± 0.6	4.0 ± 0.2	4.3 ± 0.7	5.3 ± 1.3
R_q_ [nm]	3.2 ± 0.8	3.3 ± 1.0	3.4 ± 0.7	5.2 ± 0.3	5.9 ± 1.6	6.5 ± 1.7

**Table 4:** The surface composition of the selected samples obtained by the XPS technique.

[% at.]	Non-Treated Glass	1800 s O_2_ Plasma	1800 s O_2_ Plasma + HMDS	1800 s Ar Plasma	1800 s Ar Plasma + HMDS
C 1s	19.4 ± 0.86	24.1 ± 1.30	13.3 ± 1.02	16.1 ± 1.24	10.5 ± 1.18
N 1s	0.2 ± 0.52	1.2 ± 1.06	-	2.1 ± 1.24	-
O 1s	45.5 ± 0.90	42.9 ± 1.18	48.9 ± 0.90	47.8 ± 1.34	51.4 ± 0.98
Na 1s	4.0 ± 1.23	2.3 ± 0.46	3.1 ± 0.24	1.0 ± 0.26	2.4 ± 1.24
Mg 2p	2.1 ± 0.88	2.4 ± 1.04	2.5 ± 0.72	2.0 ± 1.06	1.3 ± 0.70
Si 2p	27.1 ± 0.78	22.4 ± 0.94	29.6 ± 0.78	26.0 ± 1.05	32.9 ± 0.80
Ca 2p	1.3 ± 0.48	1.9 ± 0.34	1.5 ± 0.20	2.2 ± 0.38	1.3 ± 0.38
others	≈0.40	≈2.80	≈1.10	≈2.8	≈0.2

## Data Availability

The data presented in this study are available on request from the corresponding author.

## Supplementary Materials

The following supporting information can be downloaded at: https://www.mdpi.com/article/10.3390/molecules29112645/s1.
