# Supplementary material for: Hydrophobization of Cold Plasma Activated Glass Surfaces by Hexamethyldisilazane Treatment
Source: Molecules. 2024 Jun 4;29(11):2645. doi: 10.3390/molecules29112645 (PMC11173631; doi:10.3390/molecules29112645)
Supplement: Supplementary file 1 [file molecules-29-02645-s001.zip › molecules-2998007-supplementary.pdf]

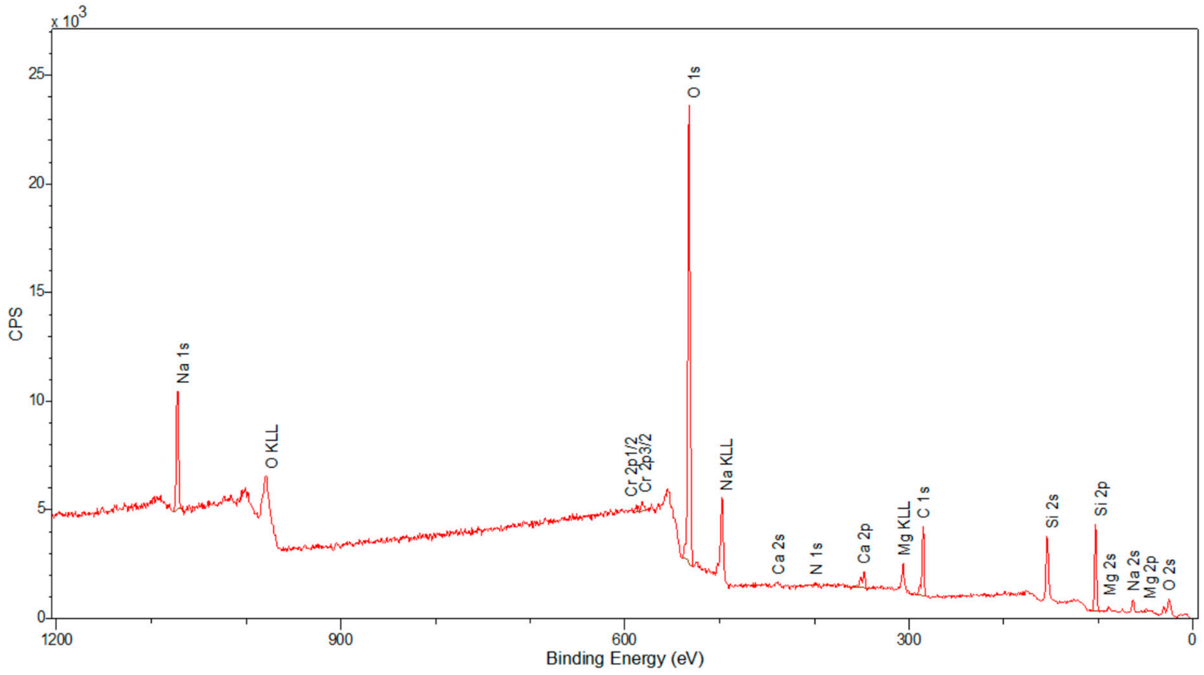

Figure S1. Wide scan energy XPS spectrum for pristine glass plate.

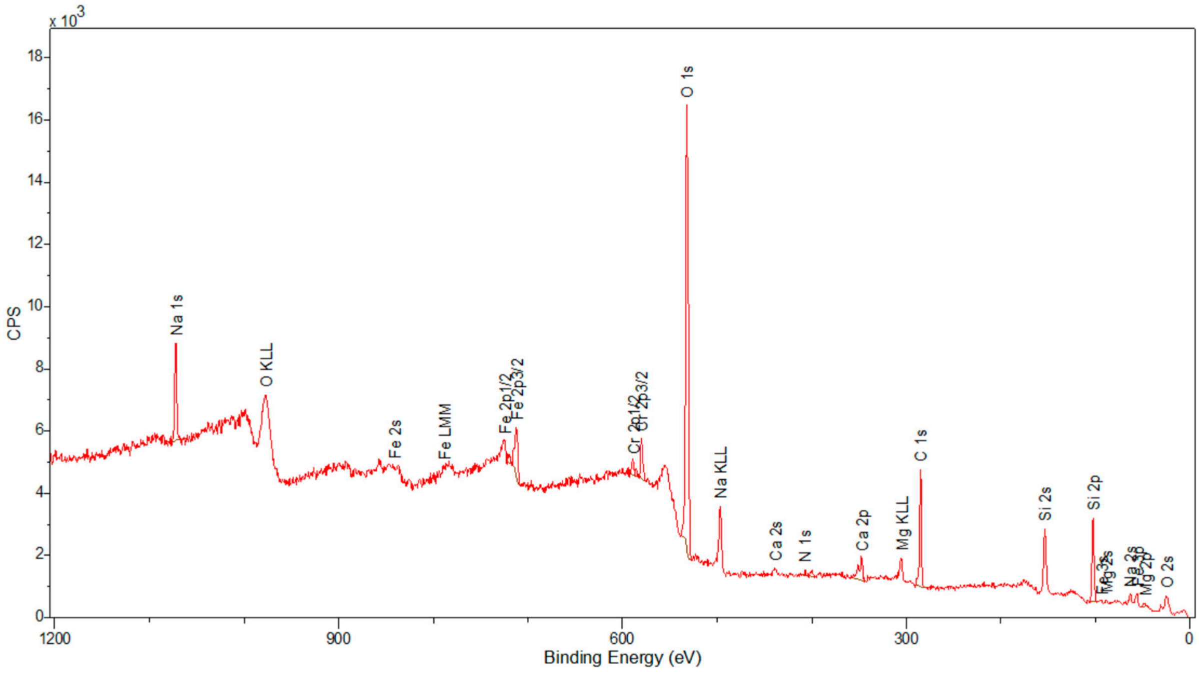

Figure S2. Wide scan energy XPS spectrum for glass plate activated 1800s by oxygen plasma.

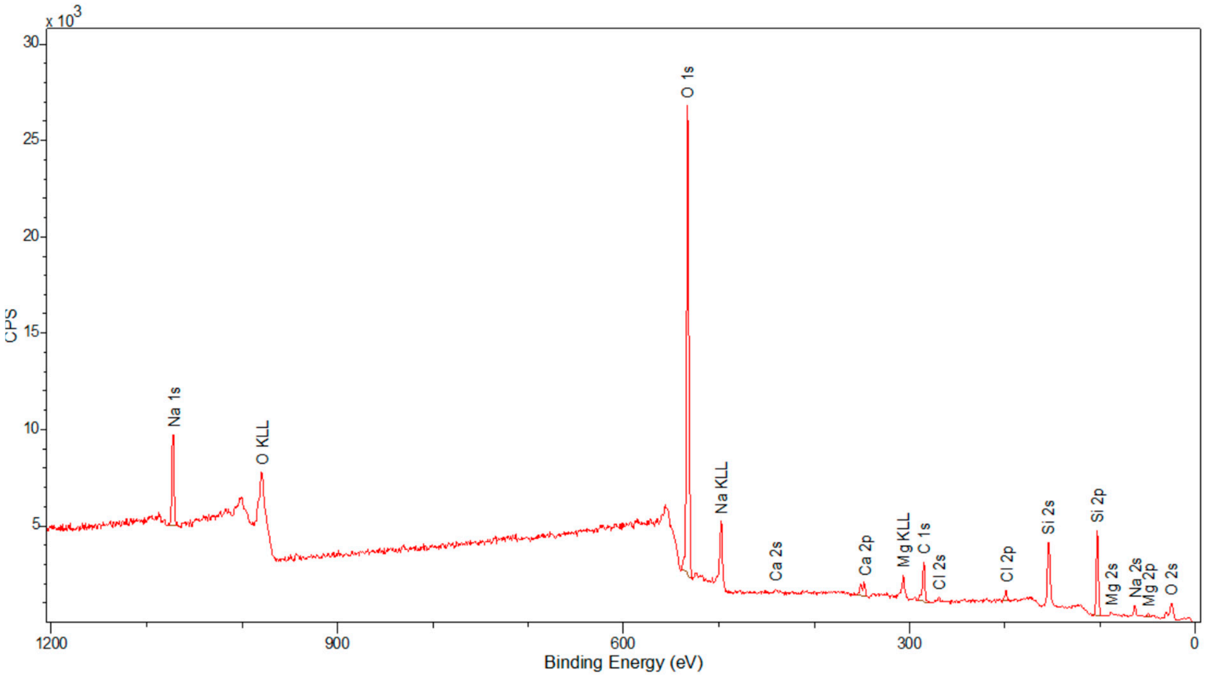

Figure S3. Wide scan energy XPS spectrum for glass plate activated 1800s by oxygen plasma and deposited HDMS.

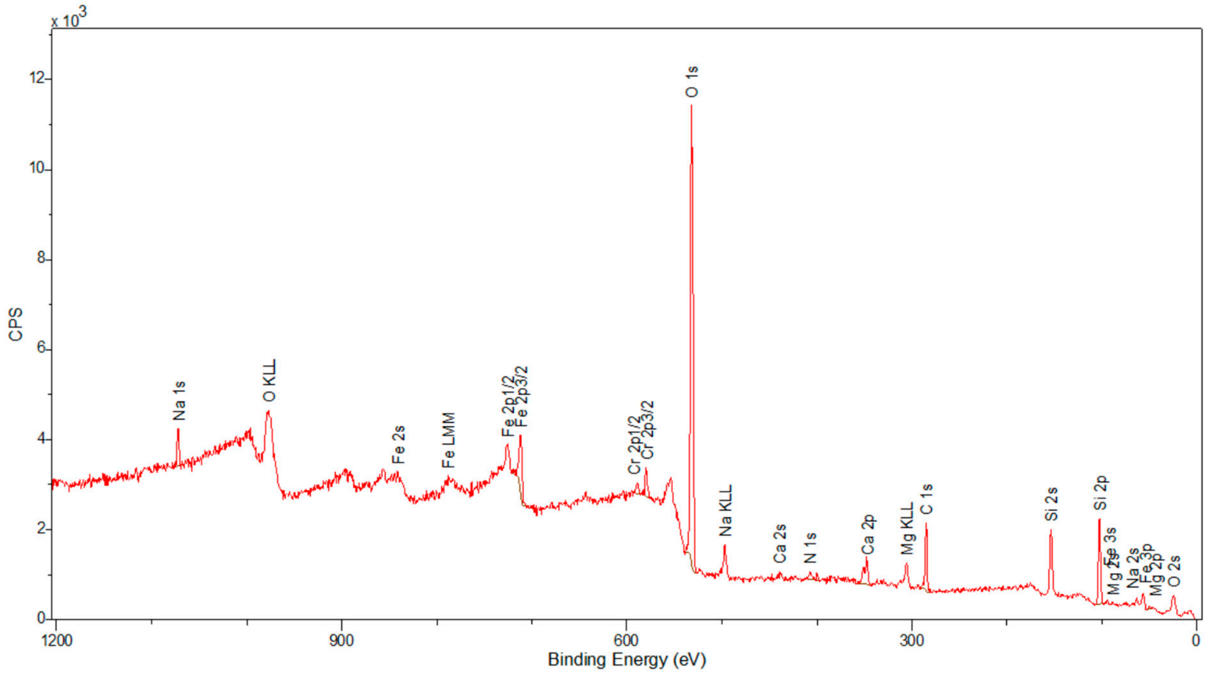

Figure S4. Wide scan energy XPS spectrum for glass plate activated 1800s by argon plasma.

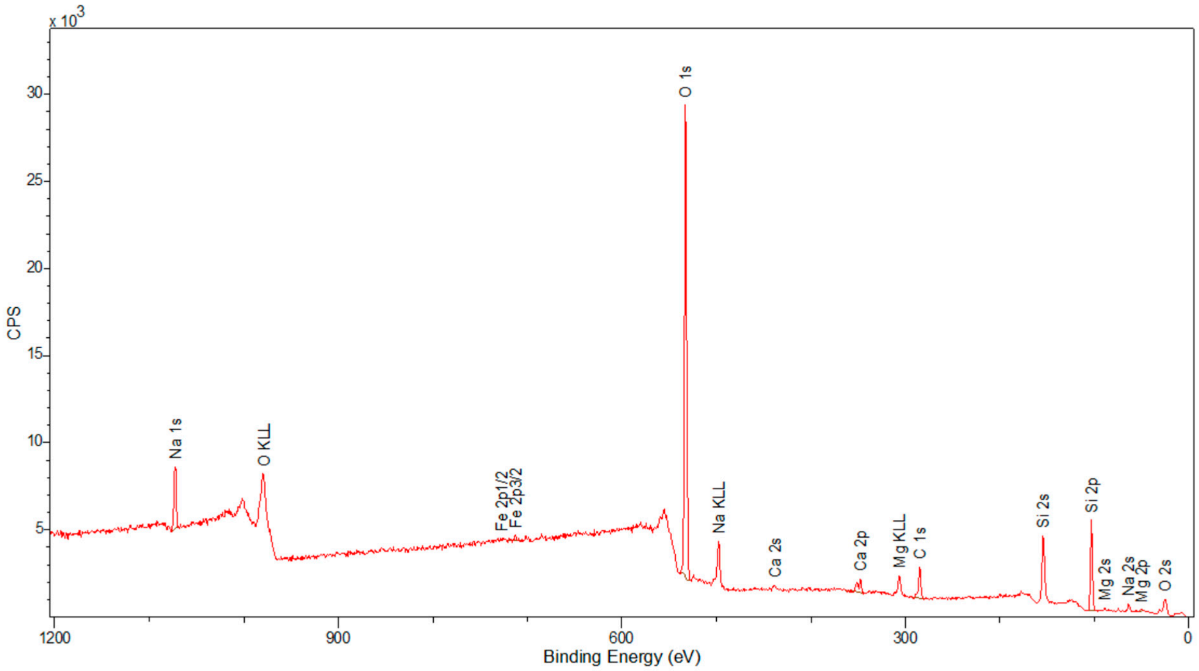

Figure S5. Wide scan energy XPS spectrum for glass plate activated 1800s by argon plasma and deposited HDMS.

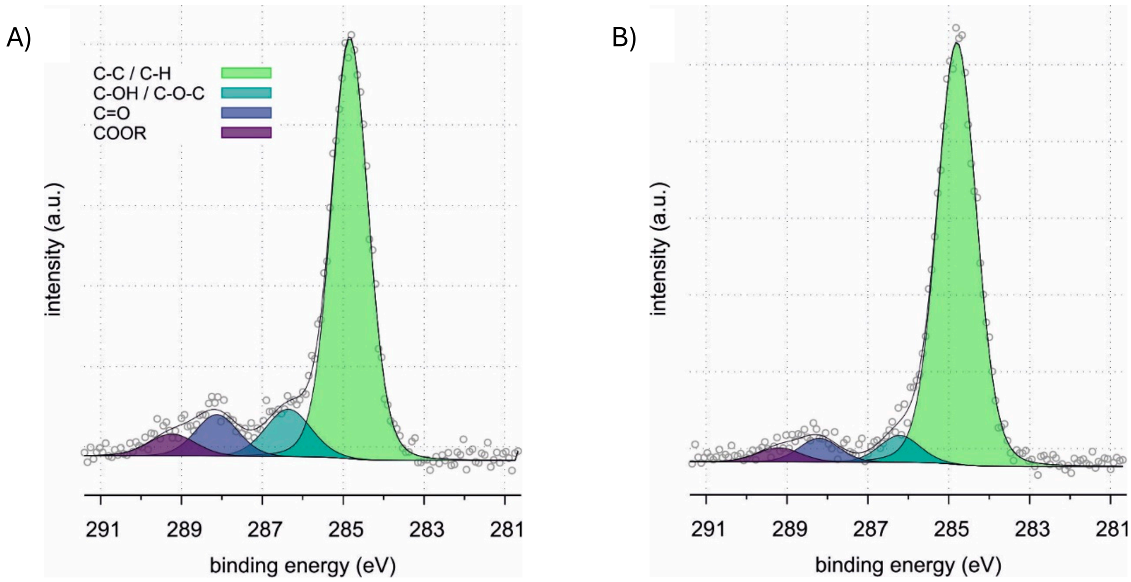

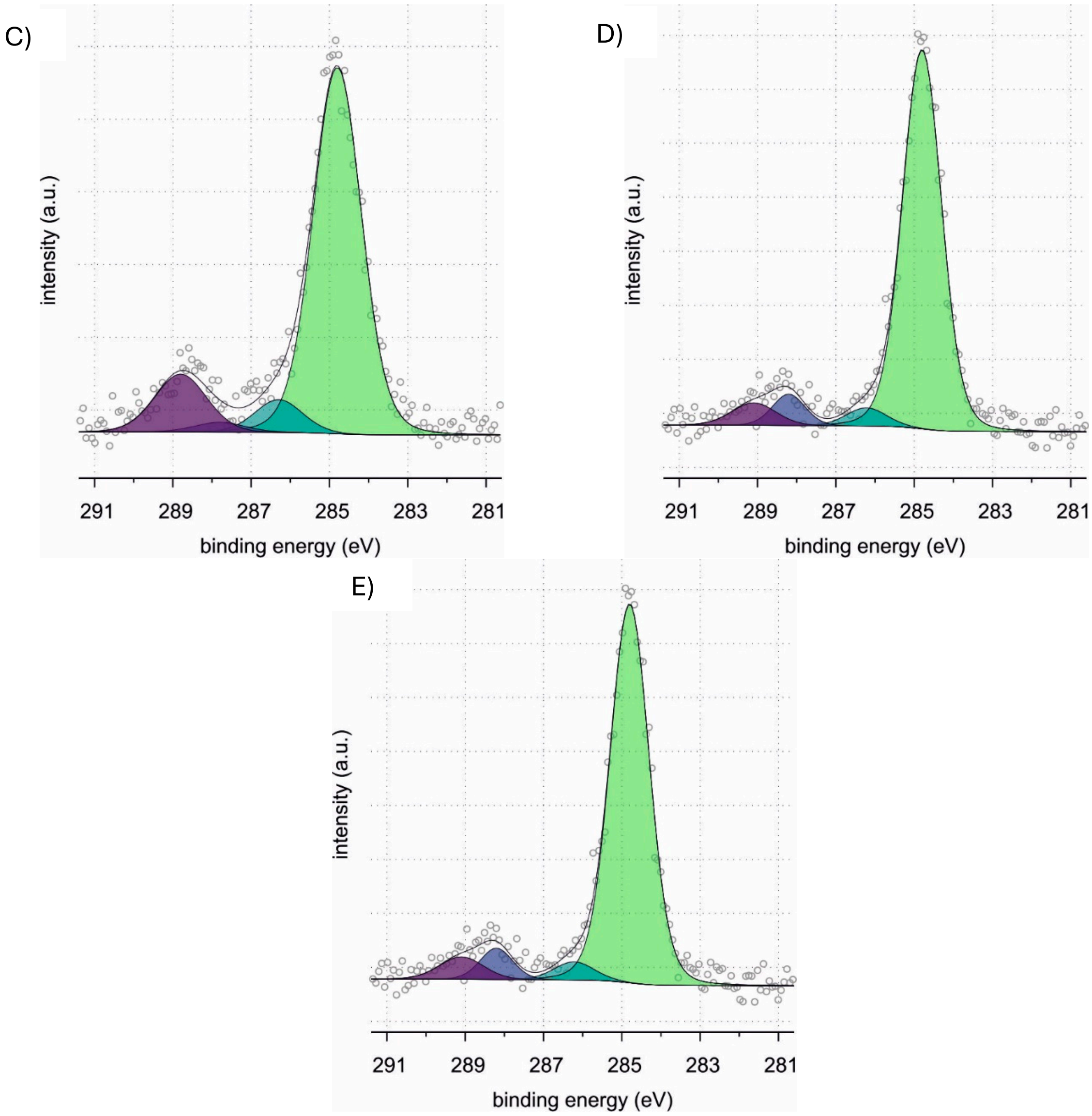

Figure S6. High-resolution deconvoluted XPS spectra for C 1s A) pristine glass B) glass activated 1800 s by oxygen plasma C) glass activated 1800 s by oxygen plasma and HDMS adsorbed D) glass activated 1800 s by argon plasma E) glass activated 1800 s by argon plasma and HDMS adsorbed

Table S1 Carbon form on tested surfaces

	pristine glass	1800 s by oxygen plasma	1800 s by oxygen plasma and HDMS adsorbed	glass activated 1800 s by argon plasma	1800 s by argon plasma and HDMS adsorbed
C-C / C-H	76.2	81.8	86.6	84.7	87.5
C-OH / C-O-C	10.4	3.8	5.5	4.1	4.9
C=O	8.5	8.4	4.8	5.7	6.2
COOR	4.9	6.0	3.1	5.5	1.1
